# Primary hexaploid synthetics: Novel sources of wheat disease resistance

**DOI:** 10.1016/j.cropro.2019.03.003

**Published:** 2019-07

**Authors:** Vladimir Shamanin, Sergey Shepelev, Violetta Pozherukova, Elena Gultyaeva, Tamara Kolomiets, Elena Pakholkova, Alexey Morgounov

**Affiliations:** a Omsk State Agrarian University, Omsk, 644008, Russia; b All-Russian Institute of Plant Protection, Pushkin, 196608, Russia; c All-Russian Phytopathology Institute, Moscow Reg, 143050, Russia; d CIMMYT, Ankara, 06511, Turkey

**Keywords:** Wheat, Rusts, Powdery mildew, *Septoria*, Genetic resistance

## Abstract

Climate change is leading to increased occurrence of and yield losses to wheat diseases. Managing these diseases by introducing new, effective and diverse resistance genes into cultivars represents an important component of sustainable wheat production. In 2016 and 2017 a set of primary hexaploid synthetic wheat was studied under high disease pressure: powdery mildew, leaf and stem rust in Omsk; *Septoria tritici* and *S. nodorum* in Moscow. A total of 28 synthetics (19 CIMMYT synthetics and 9 Japanese synthetics) were selected as having combined resistance to at least two diseases in both years of testing. Two synthetics (entries 13 and 18) originating from crosses between winter durum wheat Ukrainka odesskaya-1530.94 and various *Aegilopes taushii* accessions, and four synthetics (entries 20, 21, 23 and 24) from cross between Canadian durum wheat Langdon and *Ae. taushii* were resistant to all four pathogens. Pathological and molecular markers evaluation of resistance suggests presence of new genes and diverse types of resistance. The novel genetic sources of disease resistance identified in this study can be successfully utilized in wheat breeding.

## Highlights

• Primary synthetics demonstrated resistance to several important diseases.
• Six entries were resistant to powdery mildew, leaf/stem rust, *Septoria* in 2016/17.
• Resistance type varied from immunity to slow disease development.
• The resistance is controlled by both known and possibly new genes.
• The novel genetic sources of resistance are valuable for wheat breeding.

Wheat is a staple food crop and provides about 20 percent of protein and calories consumed per capita (CRP WHEAT, 2016). It is grown on approximately 225 million hectares worldwide with a significant portion produced as a short season crop planted in April–May and harvested in August–September in high-latitude regions above 45 °N. The western Siberia region of Russia and northern Kazakhstan cultivates 17–18 million ha of spring wheat. This region plays an important role in regional and global food security as most of the grain produced is traded. The wheat production environment, biotic and abiotic stresses, breeding system, and varieties cultivated in the region have been described by Morgounov et al. (2000).

Leaf rust is historically the major wheat disease in this area and occurs nearly every year. However, in the last five to seven years, stem rust prevalence has been increasing and caused epidemics over 1–2 million ha in 2015–2017 with estimated crop losses of 20–30 percent. *Septoria spp*. is also causing increasing damage to wheat as zero tillage technologies become more popular. Unfortunately, the majority of cultivars are susceptible to the dominant diseases and there is limited genetic diversity of resistance genes available for use in breeding programs (Shamanin et al., 2016). This study therefore aimed to identify and characterize novel sources of spring wheat resistance to major pathogens.

Wild wheat relatives have been successfully used to identify and incorporate new disease resistance genes in wheat. Moreover, synthetic hexploid wheat has recently been used as a bridge to incorporate more genomes of wild species. The most popular synthetics are based on crosses between durum wheat (*Triticum turgidum* sp. *durum*, genome AB) and *Ae. taushii* (bread wheat D genome progenitor). Synthetic wheat plants resemble the semi-wild type with tight, hardly threshable spikes and poor agronomic performance, yet they contain new diversity for resistance to numerous abiotic stresses, diseases, and pests (Ogbonnaya et al., 2013). This study utilized two groups of germplasm (Supplement 1): 1) Synthetics developed by CIMMYT from crosses between winter durum wheat varieties from Ukraine and Romania and *Ae. taushii* from the Caspian Sea basin, as described by Morgounov et al. (2018), and 2) Synthetics developed in Japan from crosses between the US durum cultivar Langdon and *Ae. taushii* selected from the global diversity collection (Matsuoka et al., 2007).

Field experiments were conducted in 2016 and 2017 at Omsk, Russia, in replicated trials with a plot size of 1 m^2^. Experiments were planted in mid-May (after fallow) in both years and were harvested in early September. Plants' reactions to powdery mildew (*Erisyphe graminis*), leaf rust (*Puccinia recondita*), and stem rust (*Puccinia graminis*) were evaluated under natural disease pressure. In both years, 4–5 severity readings were taken for each disease and the Area Under Disease Progress Curve (AUDPC) was calculated. Common agronomic traits were recorded including days to heading, plant height, and yield components. Seedlings’ reactions to leaf rust was conducted using local Chelyabinsk population of the pathogen. The presence of molecular markers to resistance genes *Lr9, Lr10, Lr19, Lr20, Lr21, Lr24, Lr26, Lr34, Lr37, Lr41, Lr67, Sr2*, and *Sr42* were evaluated using established protocols (http://maswheat.ucdavis.edu). Resistance to *Septoria* sp. was evaluated in the field under separate artificial inoculations of *S. nodorum* and *S. tritici* in the Moscow region during 2017. For all diseases, entries were classified into four main categories based on severity and AUDPC: R-resistant; MR-moderately resistant; MS-moderately susceptible; and S-susceptible (Supplement 2).

A total of 28 synthetics (19 CIMMYT synthetics and 9 Japanese synthetics) demonstrated combined resistance to at least two diseases in both years of testing (Supplement 1). Six synthetics (entries 13, 18, 20, 21, 23 and 24) were resistant to all four pathogens. Leaf rust severity and AUDPC for 2016 and 2017 is presented in Table 1. Local check variety Serebristaya showed high severity, indicating substantial disease pressure. Eleven entries demonstrated MR or R reactions across both years including six synthetics (entries 13, 14, 15, 19, 21, and 22), which possessed the *Lr41* gene, either singly or in combination with other genes. This gene derives from *Ae. taushii* (Singh et al., 2004) and is effective against Siberian rust populations. Resistant entry 7 possessed *Lr21,* which is not effective against local rusts, suggesting that there are additional genes involved. Remaining four resistant synthetics did not possess known *Lr* genes. Entry 25 (LDN/*Ae.tau.*(KU-2092)) combined resistance to leaf rust at both the seedling and adult plant stages, suggesting presence of a major gene. Synthetics 20 (LDN/*Ae.tau.*(IG-126387)), 24 (LDN/*Ae.tau.*(KU-20-9)) and 28 (LDN/*Ae.tau.*(KU-2105)) were susceptible at the seedling stage but resistant in the field, indicating the presence of possibly new adult plant resistance genes.

**Table 1:** Reaction of primary hexaploid synthetics to leaf rust, Omsk, Russia, 2016–2017.

Entry no.	Pedigree	Reaction,^a^ 2016-17	Severity,^b^ %		AUDPC,^c^cm^2^		Seedlings reaction^d^	Genes^e^
			2016	2017	2016	2017		
–	Serebristaya (Check)	S-S	80	53	1597	1248	3–4	–
1	Aisberg/*Ae.tau.*(369)	S-S	55	65	985	1392	3–4	*Lr10,Lr34*
2		MS-S	60	50	1097	999	3	*Lr10*
3		S-MS	40	55	797	954	3–4	–
4		MS-MS	30	60	862	1302	0	–
5	Aisberg/*Ae.tau.*(511)	MS-S	30	8	787	128	3	–
6		MS-R	20	20	607	507	3	*Lr10*
7	Pandur/*Ae.tau.*(223)	MR-MR	50	25	1107	464	4	*Lr21*
8		S-MR	50	30	932	717	2	*Lr41*
9	U.od.1530.94/*Ae.tau.*(1027)	MS-MS	60	35	1107	703	3	–
10		S-MS	60	23	1097	420	4	–
11		S-MR	40	35	937	852	3–4	*Lr21*
12		MS-MS	5	15	137	324	3	–
13		R-R	0	13	0	195	0	*Lr10,Lr41*
14	U.od.1530.94/*Ae.tau.*(310)	R-R	10	13	245	248	0–1	*Lr41*
15		R-R	40	15	745	290	0	*Lr34,Lr41*
16	U.od.1530.94/*Ae.tau.*(392)	MS-R	80	50	1535	964	3–4	–
17	U.od.1530.94/*Ae.tau.*(458)	S-MS	40	55	762	1128	3–4	*Lr24*
18	U.od.1530.94/*Ae.tau.*(629)	MS-S	0	8	0	160	0	–
19	U.od.952.92/*Ae.tau.*(1031)	R-R	50	15	547	261	3	*Lr41*
20	LDN/*Ae.tau.*(IG-126387)	MR-R	25	13	602	248	3	–
21	LDN/*Ae.tau.*(IG-131606)	MR-R	20	8	482	128	3–4	*Lr41*
22	LDN/*Ae.tau.*(IG-48042)	MR-R	40	8	927	128	3–4	*Lr41*
23	LDN/*Ae.tau.*(KU-2075)	MS-R	20	8	265	93	1–2	*Lr41*
24	LDN/*Ae.tau.*(KU-20-9)	R-R	30	15	535	239	3	–
25	LDN/*Ae.tau.*(KU-2092)	MR-R	40	30	807	417	0	–
26	LDN/*Ae.tau.*(KU-2093)	MS-MR	50	30	982	487	4	–
27	LDN/*Ae.tau.*(KU-2096)	MS-MR	10	8	182	130	3	–
28	LDN/*Ae.tau.*(KU-2105)	R-R	50	25	1317	364	4	–

a R-Resistant. MR- Moderately resistant, MS – Moderately susceptible, S- susceptible. First reading for 2016, 2nd reading for 2017.

b Highest severity recorded during the season.

c Area under disease progress curve calculated using 5 reading in 2016 and 4 readings in 2017.

d Seedlings reaction (0; 1; 2 – resistant, 3; 4 – susceptible) to Chelyabinsk leaf rust population (Virulence/avirulence: 1,2a,2b,2c,3a,3bg,3ka,9,10,11,14a,14b,15,16, 17,18,20,21, 30/19,23,24,26,29) conducted by All-Russian Crop Protection Institute (St. Petersburg).

e Molecular markers were used for identification of the following genes*: Lr9*, *Lr10*, *Lr19*, *Lr20*, *Lr21*, *Lr24*, *Lr26*, *Lr34*, *Lr37*, *Lr41, Lr67*.

Most of the synthetics (21 of 28) were resistant to stem rust under high disease pressure, while severity on the local check exceeded 80% in 2016 and 40% in 2017 (Table 2). Near immunity was recorded for entries 5 (Aisberg/*Ae.tau*.(511)), 9 (U.od.1530.94/*Ae.tau.*(1027)), 18 (U.od.1530.94/*Ae.tau.*(629)), and 26 (LDN/*Ae.tau*.(KU-2093)). The study only evaluated the presence of two genes: *Sr2* and *Sr42*. None of the genotypes possessed gene *Sr2*. Eight synthetics carried gene *Sr42* including six resistant to stem rust in both years. This gene is located on chromosome 6DS provides resistance to the Ug99 stem rust race TTKSK (Gao et al., 2015). Obviously, it migrated to the synthetics from *Ae. taushii*. Evaluation of the stem rust trap nursery demonstrated that the *Sr42* gene is not as effective in Siberia, with severity reaching 40MS. However, it may contribute to reduced severity of stem rust. Variety Langdon, which was used as a durum parent in Japanese synthetics, is known to possess *Sr9e* (Luig, 1993). This gene provides intermediate protection under Siberian conditions with severity 10MS-20S. So high frequency of stem rust resistant synthetics is explained by contribution of genes from both parents. A recent study of stem rust resistant material in Siberia (Shamanin et al., 2016) demonstrated a very narrow genetic basis of resistance. Given that the majority of spring wheat cultivars from Siberia and Kazakhstan are highly susceptible to stem rust, these new, resistant synthetics offer valuable parental material.

**Table 2:** Reaction of primary hexaploid synthetics to stem rust and powdery mildew, Omsk, Russia, 2016–2017.

Entry no.	Pedigree	Stem rust						Powdery mildew				
		Reaction, 2016-17	Severity,^a^ %		AUDPC^b^,cm^2^		Genes	Reaction, 2016-17	Severity^a^, %		AUDPC^b^,cm^2^	
			2016	2017	2016	2017			2016	2017	2016	2017
–	Serebristaya (Check)	S-S	80	40	1011	725	–	S-S	80	70	1789	1870
1	Aisberg/*Ae.tau.*(369)	MR-MR	50	15	478	207	–	R-R	0	20	0	367
2		MR-MS	40	18	448	320	–	R-MR	10	30	267	737
3		MR-R	40	5	375	67	–	R-MR	0	30	0	705
4		MR-R	30	5	330	84	–	R-R	10	10	267	277
5	Aisberg/*Ae.tau.*(511)	R-R	0	10	0	102	–	MR-S	30	85	807	2112
6		MR-MS	20	20	418	347	*Sr42*	MR-MS	30	70	807	1382
7	Pandur/*Ae.tau.*(223)	MR-R	30	5	314	49	*Sr42*	R-MR	10	35	267	677
8		MS-R	50	5	613	67	–	R-R	50	10	447	277
9	U.od.1530.94/*Ae.tau.*(1027)	R-R	15	8	213	128	–	MS-MR	70	50	1087	987
10		MR-MR	30	20	275	277	–	R-MS	10	65	217	1237
11		MR-MS	40	25	400	347	–	MR-S	30	70	557	1587
12		S-MS	70	35	1048	574	–	R-R	0	10	0	277
13		MR-R	50	13	448	180	–	R-R	0	10	0	277
14	U.od.1530.94/*Ae.tau.*(310)	MS-MR	60	13	608	226	–	R-MR	0	35	0	790
15		MR-R	20	8	255	128	–	MR-MR	30	25	557	552
16	U.od.1530.94/*Ae.tau.*(392)	R-R	20	5	230	49	*Sr42*	R-R	10	20	142	482
17	U.od.1530.94/*Ae.tau.*(458)	R-R	20	13	245	163	–	S-MR	80	30	1782	687
18	U.od.1530.94/*Ae.tau.*(629)	R-R	5	13	60	180	–	R-R	10	10	267	277
19	U.od.952.92/*Ae.tau.*(1031)	MR-MR	30	15	389	207	*Sr42*	R-MR	10	40	217	935
20	LDN/*Ae.tau.*(IG-126387)	MR-R	50	5	502	49	–	R-MR	10	30	267	762
21	LDN/*Ae.tau.*(IG-131606)	MR-R	40	8	425	110	–	MR-MR	30	50	557	927
22	LDN/*Ae.tau.*(IG-48042)	R-R	20	3	200	23	*Sr42*	R-MR	0	30	0	687
23	LDN/*Ae.tau.*(KU-2075)	R-R	20	5	173	49	*Sr42*	R-MR	10	20	142	545
24	LDN/*Ae.tau.*(KU-20-9)	MR-R	50	3	373	23	–	MR-MR	50	30	627	687
25	LDN/*Ae.tau.*(KU-2092)	MS-R	60	0	605	0	*Sr42*	R-R	10	10	217	277
26	LDN/*Ae.tau.*(KU-2093)	R-R	10	5	98	49	–	R-R	0	10	0	277
27	LDN/*Ae.tau.*(KU-2096)	MR-R	30	5	275	49	–	S-MR	90	40	2027	780
28	LDN/*Ae.tau.*(KU-2105)	MR-R	50	10	450	102	*Sr42*	R-R	10	10	267	277

a Highest severity recorded during the season.

b Area under disease progress curve calculated using 5 reading in 2016 and 3 readings in 2017 for stem rust and, respectively, 4 and 4 in for powdery mildew.

A high level of resistance to powdery mildew is not common in bread wheat. A total of 21 synthetics demonstrated R or MR reactions to powdery mildew in both years, including nine that were practically immune: entries 1 and 4 (Aisberg/*Ae.tau*.(369)); 8 (Pandur/*Ae.tau.*(223)); 12 and 13 (U.od.1530.94/*Ae.tau*.(1027)); 16 (U.od.1530.94/*Ae.tau*.(392)); 18 (U.od.1530.94/*Ae.tau*.(629)); and 25, 26, and 28 (based on durum wheat Langdon). For most of these, powdery mildew severity did not exceed 10–20% and AUDPC was below 400, while the corresponding figures for the susceptible check exceeded 70% and 1,700. *Ae. tauschii* has proved to be a valuable source of powdery mildew resistance, providing genes *Pm2*, *Pm10*, *Pm15*, *Pm19*, *Pm34, Pm35* (Alam et al., 2011) and *Pm58* (Wiersma et al., 2017). Durum wheat is a less valuable source of powdery mildew resistance, though it contributed gene *Pm3h*. The presence of *Pm* genes has not been evaluated in this germplasm but deserves attention in the future.

Resistance to *Septoria tritici* was evaluated under artificial field inoculation near Moscow in 2017. The susceptible check was completely defeated by *S. tritici*, with a severity of 100%. Most CIMMYT synthetics demonstrated MR reactions with 5–10% flag leaf severity and severity of the 2nd and 3rd leaves up to 40% (Table 3). All Japanese synthetics demonstrated R reactions with 5% flag leaf severity and 20–30% 2nd and 3rd leaf severity. Reactions to *S. tritici* were also evaluated at the seedling stage under artificial inoculation by recording severity and number of spores per leaf. There was no significant correlation between disease severity on seedlings and adult plant leaves. However, the correlation between field severity and number of spores per leaf was significant and varied from 0.49 (seedlings – 2nd and 3rd leaves) to 0.56 (seedlings – flag leaf). Across the traits used to evaluate reactions to *S. tritici*, (field and seedling severity, number of spores per leaf) the following synthetics demonstrated superior resistance: 1 and 2 (Aisberg/Ae.tau.(369)), 8 (Pandur/Ae.tau.(223)), 13 (U.od.1530.94/Ae.tau.(1027)), 15 (U.od.1530.94/Ae.tau.(310)), 18 (U.od.1530.94/Ae.tau.(629)), 24 (LDN/Ae.tau.(KU-20-9)), 25 (LDN/Ae.tau.(KU-2092)), and 28 (LDN/Ae.tau.(KU-2105)). More than 20 *S. tritici* resistance genes have been identified in wheat, including *Stb5*, *Stb8*, *Stb16q,* and *Stb17* (which originated from synthetic wheat) (Brown et al., 2015). Gene *Stb16q* – located on chromosome 3DL and originating from *Ae. taushii* accession C122 – provided a high level of resistance to global isolates at both the seedling and adult plant stages (Ghaffary et al., 2012). The type of resistance to *S. tritici* identified in the above listed synthetics was similar to *Stb16q* though specific study is needed to prove it.

**Table 3:** Reaction of primary hexaploid synthetics to *Septoria* sp., Moscow region, Russia, 2017.

Entry no.	Pedigree	Reaction, 2017	Field artificial inoculation severity,^a^ %			Seedlings artificial inoculation		
			2^nd^-3rd leaves	Flag leaf	Spike	Severity (%): *S. nodorum*	Severity (%): *S. tritici*	Spores^b^/leaf: *S.tritici*
–	OMGAU-90 (Check)	S	100	100	60	56.5	33.7	223.4
1	Aisberg/*Ae.tau.*(369)	MR	40	5	10	66.5	8.8	5.2
2		MR	40	5	20	44.3	8.0	5.2
3		MR	40	10	10	34.0	26.0	7.8
4		MR	40	5	25	35.7	26.7	6.7
5	Aisberg/*Ae.tau.*(511)	MS	40	50	50	70.0	19.7	5.2
6		MS	20	80	40	47.0	22.2	19.1
7	Pandur/*Ae.tau.*(223)	MS	60	5	25	9.5	6.0	1.5
8		MR	40	10	40	35.8	0.0	0.0
9	U.od.1530.94/*Ae.tau.*(1027)	MR	40	10	40	29.0	9.0	21.8
10		MR	40	5	20	13.0	41.2	68.7
11		MR	40	5	20	41.5	7.7	15.6
12		MR	40	5	10	25.0	21.2	23.4
13		R	20	5	25	75.4	9.5	10.9
14	U.od.1530.94/*Ae.tau.*(310)	MS	60	20	40	25.5	10.8	45.1
15		MR	40	5	15	3.1	0.4	0.0
16	U.od.1530.94/*Ae.tau.*(392)	MS	60	5	20	69.0	11.8	29.3
17	U.od.1530.94/*Ae.tau.*(458)	MR	40	10	20	59.5	23.3	46.8
18	U.od.1530.94/*Ae.tau.*(629)	MR	40	5	10	13.7	9.0	7.8
19	U.od.952.92/*Ae.tau.*(1031)	MS	60	10	50	43.2	3.0	0.0
20	LDN/*Ae.tau.*(IG-126387)	R	20	5	10	35.2	13.2	2.2
21	LDN/*Ae.tau.*(IG-131606)	R	20	5	30	17.1	9.7	17.2
22	LDN/*Ae.tau.*(IG-48042)	R	20	5	25	87.2	52.5	6.2
23	LDN/*Ae.tau.*(KU-2075)	R	20	5	40	100.0	58.3	64.2
24	LDN/*Ae.tau.*(KU-20-9)	R	20	5	30	32.0	6.3	1.4
25	LDN/*Ae.tau.*(KU-2092)	R	30	5	20	32.2	10.9	0.0
26	LDN/*Ae.tau.*(KU-2093)	R	20	5	15	37.2	26.1	13.8
27	LDN/*Ae.tau.*(KU-2096)	R	20	0	20	69.9	20.5	120.3
28	LDN/*Ae.tau.*(KU-2105)	R	20	5	20	39.2	2.5	0.0

a Inoculated by *S. nodorum* and *S. trtici.*

b The values in 1000 spores.

For the susceptible check, spike disease severity due to *S. nodorum* reached 60%, while it ranged from 10 to 40% for the resistant genotypes, with MR or R leaf reactions. The correlation between flag leaf and spike severity was significant and reached 0.65, while the correlation between spike severity and seedlings inoculated by *S. nodorum* was insignificant (0.25). Only two genotypes combined *S. nodorum* resistance on spikes and seedlings: 18 (U.od.1530.94/*Ae.tau*.(629)) and 15 (U.od.1530.94/*Ae.tau*.(310)). Individual genes have less of an effect on the resistance reactions to *S. nodorum,* compared *S. tritici,* and toxins play an important role (Oliver et al., 2008). Identifying and utilizing novel sources of resistance is a vital part of controlling *Septoria sp*.

Synthetics’ agronomic traits were evaluated in a replicated yield trial during 2017 (Supplement 3). While several entries headed earlier than the check variety Serebristaya, the majority of synthetics headed 5–12 days later than the check. Synthetic germplasm generally displayed shorter plant height and a longer spike. Spike fertility in CIMMYT synthetics, expressed by the number of grains/spike, was comparable to the check, while the Japanese synthetics had substantially lower spike fertility. Several entries displayed very large grains, with 1000 kernel weight reaching 52–57 g, or 15–20% higher than the check. All the synthetics tested, especially the Japanese material, had lower grain yield per unit area than the check.

Climate change is leading to increased occurrence of and yield losses to wheat diseases, especially in high rainfall environments. The current practices of relying on chemical protection with little contribution of genetic resistance is not sustainable. Managing these diseases by introducing new, effective and diverse resistance genes into cultivars represents an important component of sustainable wheat production. The novel genetic sources of disease resistance identified in this study can be successfully utilized in breeding to reach this goal. However, considering their poor agronomic adaption and grain yield, using these synthetics in back- or top-crosses is more likely to result in competitive products. Seeds of all germplasm presented here are available upon request.
