# Supplementary material for: Primary hexaploid synthetics: Novel sources of wheat disease resistance
Source: Crop Prot. 2019 Jul;121:7–10. doi: 10.1016/j.cropro.2019.03.003 (PMC6559260; doi:10.1016/j.cropro.2019.03.003)
Supplement: Multimedia component 1 [file mmc1.docx]

Supplement 1. Summary reaction of primary hexaploid synthetics to diseases in Russia, 2016-2017.

| Entry no. | 2017 plot ID | Pedigree | *Aegilops taushii* origin | Reaction to diseases^a^: | | | |
| --- | --- | --- | --- | --- | --- | --- | --- |
|  |  |  |  | Leaf Rust | Stem Rust | Powdery Mildew | *Septoria* sp. |
| - | - | Serebristaya (Check) | - | S-S | S-S | S-S | S |
| 1 | 31 | Aisberg/*Ae.tau.*(369) | Mazandaran, Iran | MS-S | MR-MR | R-R | MR |
| 2 | 18 |  |  | S-MS | MR-MS | R-MR | MR |
| 3 | 32 |  |  | MS-MS | MR-R | R-MR | MR |
| 4 | 38 |  |  | MS-S | MR-R | R-R | MR |
| 5 | 24 | Aisberg/*Ae.tau.*(511) | Unknown | MS-R | R-R | MR-S | MS |
| 6 | 55 |  |  | MR-MR | MR-MS | MR-MS | MS |
| 7 | 26 | Pandur/*Ae.tau.*(223) | Gilan, Iran | S-MR | MR-R | R-MR | MS |
| 8 | 61 |  |  | MS-MS | MS-R | R-R | MR |
| 9 | 41 | Ukr.od.1530.94/*Ae.tau*(1027) | Mazandaran, Iran | S-MS | R-R | MS-MR | MR |
| 10 | 45 |  |  | S-MR | MR-MR | R-MS | MR |
| 11 | 62 |  |  | MS-MS | MR-MS | MR-S | MR |
| 12 | 49 |  |  | R-R | S-MS | R-R | MR |
| 13 | 19 |  |  | R-R | MR-R | R-R | R |
| 14 | 42 | Ukr.od.1530.94/*Ae.tau.*(310) | Gilan, Iran | R-R | MS-MR | R-MR | MS |
| 15 | 37 |  |  | MS-R | MR-R | MR-MR | MR |
| 16 | 57 | Ukr.od.1530.94/*Ae.tau.*(392) | Shamahi, Azerbaijan | S-MS | R-R | R-R | MS |
| 17 | 6 | Ukr.od.1530.94/*Ae.tau.*(458) | Unknown | MS-S | R-R | S-MR | MR |
| 18 | 35 | Ukr.od.1530.94/*Ae.tau.*(629) | Mazandaran, Iran | R-R | R-R | R-R | MR |
| 19 | 9 | Ukr.od.952.92/*Ae.tau.*(1031) | Zanjan, Iran | MR-R | MR-MR | R-MR | MS |
| 20 | 29 | Langdon/*Ae.tau.*(IG-126387) | Ashkhabad, Turkmenistan | MR-R | MR-R | R-MR | R |
| 21 | 51 | Langdon/*Ae.tau.*(IG-131606) | Talas, Kyrgyzstan | MR-R | MR-R | MR-MR | R |
| 22 | 22 | Langdon/*Ae.tau.*(IG-48042) | Jammu& Kashmir | MS-R | R-R | R-MR | R |
| 23 | 14 | Langdon/*Ae.tau.*(KU-2075) | Behshahr, Iran | R-R | R-R | R-MR | R |
| 24 | 21 | Langdon/*Ae.tau.*(KU-20-9) |  | MR-R | MR-R | MR-MR | R |
| 25 | 63 | Langdon/*Ae.tau.*(KU-2092) | Babulsar, Iran | MS-MR | MS-R | R-R | R |
| 26 | 47 | Langdon/*Ae.tau.*(KU-2093) |  | MS-MR | R-R | R-R | R |
| 27 | 5 | Langdon/*Ae.tau.*(KU-2096) |  | R-R | MR-R | S-MR | R |
| 28 | 65 | Langdon/*Ae.tau.*(KU-2105) | Pahlavi, Iran | S-MR | MR-R | R-R | R |

^a^ – R-Resistant. MR- Moderately resistant, MS – Moderately susceptible, S- susceptible. First reading for 2016, 2^nd^ reading for 2017. *Septoria* sp. reaction was evaluated only in 2017.

Supplement 2. Classification of synthetics lines into disease reaction category for rusts, powdery mildew and *Septoria* based on severity and AUDPC.

| Disease | Parameter | Disease reaction category | | | | | | | |
| --- | --- | --- | --- | --- | --- | --- | --- | --- | --- |
|  |  | R | | MR | | MS | | S | |
|  |  | 2016 | 2017 | 2016 | 2017 | 2016 | 2017 | 2016 | 2017 |
| Leaf rust | Severity, % | <20 | | 20-40 | | 41-60 | | >60 | |
|  | AUDPC | <350 | | 350-700 | | 701-1050 | | >1051 | |
| Leaf rust | Severity, % | <25 | <15 | 26-50 | 16-20 | 51-60 | 21-30 | >60 | >30 |
|  | AUDPC | <250 | <200 | 250-500 | 201-400 | 501-750 | 401-600 | >750 | >600 |
| Powdery mildew | Severity, % | <30 | | 31-50 | | 51-70 | | >70 | |
|  | AUDPC | <500 | | 501-1000 | | 1001-1500 | | >1500 | |
| *Septoria* | Field severity 2^nd^-3^rd^ leaves, % | - | 20-30 | - | 40 | - | 60 | - | 100 |
|  | Field severity flag leaf, % | - | 5 | - | 5-10 | - | 5-80 | - | 100 |

Supplement 3. Main agronomic traits of primary hexaploid synthetics, Omsk, Russia, 2017.

| Entry no. | Pedigree | Days to heading^a^ | Plant height, cm | Spike length, cm | Grains/  spike | 1000 KW, gr | Grain yield, gr/m^2^ |
| --- | --- | --- | --- | --- | --- | --- | --- |
| - | Serebristaya (Check) | 40.1 | 84.7 | 6.9 | 29.4 | 44.8 | 376 |
| 1 | Aisberg/*Ae.tau.*(369) | 33.5 | 81.8 | 8.6 | 32.9 | 46.1 | 180 |
| 2 |  | 44.5 | 81.3 | 9.5 | 36.9 | 49.3 | 301 |
| 3 |  | 56.0 | 75.9 | 9.4 | 37.5 | 46.1 | 304 |
| 4 |  | 47.0 | 71.4 | 9.0 | 35.2 | 47.4 | 238 |
| 5 | Aisberg/*Ae.tau.*(511) | 31.0 | 72.8 | 7.9 | 24.4 | 50.5 | 132 |
| 6 |  | 35.0 | 74.9 | 8.6 | 21.8 | 52.4 | 170 |
| 7 | Pandur/*Ae.tau.*(223) | 53.0 | 73.5 | 9.3 | 28.6 | 49.8 | 103 |
| 8 |  | 52.0 | 74.7 | 8.7 | 31.6 | 50.1 | 311 |
| 9 | U.od.1530.94/*Ae.tau.*(1027) | 46.5 | 66.1 | 7.1 | 19.2 | 41.4 | 126 |
| 10 |  | 45.0 | 78.9 | 7.6 | 26.9 | 45.0 | 212 |
| 11 |  | 47.0 | 75.8 | 9.8 | 29.7 | 45.5 | 221 |
| 12 |  | 44.5 | 74.4 | 6.6 | 22.5 | 43.9 | 145 |
| 13 |  | 42.0 | 72.1 | 8.3 | 31.9 | 36.2 | 100 |
| 14 | U.od.1530.94/*Ae.tau.*(310) | 45.5 | 74.0 | 8.4 | 29.3 | 39.4 | 147 |
| 15 |  | 47.5 | 73.6 | 8.4 | 28.0 | 48.8 | 188 |
| 16 | U.od.1530.94/*Ae.tau.*(392) | 56.5 | 77.6 | 9.4 | 35.1 | 52.2 | 201 |
| 17 | U.od.1530.94/*Ae.tau.*(458) | 53.0 | 78.1 | 7.8 | 32.8 | 42.7 | 174 |
| 18 | U.od.1530.94/*Ae.tau.*(629) | 54.5 | 65.2 | 8.3 | 33.0 | 33.0 | 59 |
| 19 | U.od.952.92/*Ae.tau.*(1031) | 53.0 | 70.2 | 5.7 | 28.3 | 45.8 | 163 |
| 20 | LDN/*Ae.tau.*(IG-126387) | 57.0 | 69.3 | 9.2 | 27.1 | 45.5 | 103 |
| 21 | LDN/*Ae.tau.*(IG-131606) | 57.0 | 64.6 | 9.9 | 20.8 | 41.2 | 61 |
| 22 | LDN/*Ae.tau.*(IG-48042) | 54.5 | 78.9 | 10.2 | 26.2 | 43.9 | 138 |
| 23 | LDN/*Ae.tau.*(KU-2075) | 52.0 | 67.6 | 10.0 | 18.2 | 35.6 | 46 |
| 24 | LDN/*Ae.tau.*(KU-20-9) | 49.5 | 81.6 | 7.7 | 21.2 | 57.4 | 83 |
| 25 | LDN/*Ae.tau.*(KU-2092) | 49.5 | 66.5 | 7.1 | 24.0 | 53.4 | 35 |
| 26 | LDN/*Ae.tau.*(KU-2093) | 53.0 | 82.3 | 9.9 | 22.5 | 43.7 | 37 |
| 27 | LDN/*Ae.tau.*(KU-2096) | 49.0 | 52.8 | 9.8 | 15.7 | 44.2 | 59 |
| 28 | LDN/*Ae.tau.*(KU-2105) | 50.0 | 70.6 | 9.9 | 21.7 | 49.5 | 99 |
|  | LSD | 2.6 | 2.6 | 0.5 | 2.2 | 2.1 | 34 |

^a^ – from crop emergence.
